# A hybrid model for tuberculosis forecasting based on empirical mode decomposition in China

**DOI:** 10.1186/s12879-023-08609-x

**Published:** 2023-10-07

**Authors:** Ruiqing Zhao, Jing Liu, Zhiyang Zhao, Mengmeng Zhai, Hao Ren, Xuchun Wang, Yiting Li, Yu Cui, Yuchao Qiao, Jiahui Ren, Limin Chen, Lixia Qiu

**Affiliations:** 1 Department of Health Statistics, School of Public Health, Shanxi Medical University, Taiyuan, Shanxi China; 2 School of Public Health, Sun Yat-Sen University, Guangzhou, China; 3 Shanxi Provincial Peoples Hospital, Taiyuan City, Shanxi Province, China

**Keywords:** Forecasting, Empirical mode decomposition, Hybrid model, Time series analysis, Tuberculosis

## Abstract

**Background:**

Pulmonary Tuberculosis is a major public health problem endangering people's health, a scientifically accurate predictive model is of great practical significance for the prevention and treatment of pulmonary tuberculosis.

**Methods:**

The reported incidence data of pulmonary tuberculosis were from the National Public Health Science Data Center (https://www.phsciencedata.cn/). The ARIMA, LSTM, EMD-SARIMA, EMD-LSTM, EMD-ARMA-LSTM models were established using the reported monthly incidence of tuberculosis reported in China from January 2008 to December 2018. The MSE, MAE, RMSE and MAPE were used to evaluate the performance of the models to determine the best model.

**Results:**

Comparing decomposition-based single model with undecomposed single model, it was found that: when predicting the incidence trend in the next year, compared with SARIMA model, the MSE, MAE, RMSE and MAPE of EMD-SARIMA decreased by 39.3%, 19.0%, 22.1% and 19.8%, respectively. The MSE, MAE, RMSE and MAPE of EMD-LSTM were reduced by 40.5%, 12.8%, 22.9% and 12.7%, respectively, compared with the LSTM model; Comparing the decomposition-based hybrid model with the decomposition-based single model, it was found that: when predicting the incidence trend in the next year, compared with EMD-SARIMA model, the MSE, MAE, RMSE and MAPE of EMD-ARMA-LSTM model decreased by 21.7%, 10.6%, 11.5% and 11.2%, respectively. The MSE, MAE, RMSE and MAPE of EMD-ARMA-LSTM were reduced by 16.7%, 9.6%, 8.7% and 12.3%, respectively, compared with EMD-LSTM model. Furthermore, the performance of the model were consistent when predicting the incidence trend in the next 3 months, 6 months and 9 months.

**Conclusion:**

The prediction performance of the decomposition-based single model is better than that of the undecomposed single model, and the prediction performance of the combined model using the advantages of different models is better than that of the decomposition-based single model, so the EMD-ARMA-LSTM combination model can improve the prediction accuracy better than other models, which can provide a theoretical basis for predicting the epidemic trend of pulmonary tuberculosis and formulating prevention and control policies.

## Introduction

Tuberculosis (TB) is a contagious disease caused by infection with the bacterium Mycobacterium tuberculosis [1], which is spread when people who are sick with TB expel bacteria into the air (e.g. by coughing). TB typically affects the lungs (pulmonary TB) but can also affect other sites (extrapulmonary TB). China is one of the 30 countries with a high burden of TB in the World. The World Health Organization (WHO) estimated in its Global Tuberculosis Report 2021 that the number of new TB cases in China in 2020 would be 842,000 (833,000 in 2019). The TB incidence rate was 59 cases per 100,000 population per year (58/100,000 in 2019), accounting for 8.5% (8.4% in 2019) of the global total and the second highest after India [2]. In our country, pulmonary tuberculosis belongs to class B legal reportable infectious diseases, and its reported incidence number always ranks in the forefront of class A and B notifiable infectious diseases nationwide. Actually, in recent years, Since the extensive use of anti-tuberculosis drugs such as isoniazid and rifampin and the government-led mobilization of the whole society, pulmonary tuberculosis has been effectively controlled to a certain extent, and the incidence of tuberculosis has also shown a trend of decreasing year by year. Nevertheless the spread of Drug-Resistant Tuberculosis bacilli makes the situation of drug-resistant tuberculosis (DR-TB) not optimistic [3], which further aggravates the public health threat to tuberculosis control. With more and more challenges in the prevention and treatment of pulmonary tuberculosis, the prediction of its incidence has become a hot topic. It is of great practical significance to explore the trend and regularity of pulmonary tuberculosis and establish a scientifically accurate predictive model for the prevention and treatment of pulmonary tuberculosis.

The data of infectious diseases changing over time are random, but generally show an upward or downward trend, which makes it possible to predict the incidence trend, but it is still difficult to make accurate prediction [4]. Many scholars have predicted the trend of infectious diseases based on historical data, The relatively perfect and accurate algorithms for the analysis and prediction of time series data mainly include Autoregressive Integrated Moving Average (ARIMA) model based on traditional statistical method and Long-Short term Memory neural network (LSTM) model based on neural network method. Both ARIMA model and LSTM model can well predict the future data according to the laws extracted from the original data. However, The ARIMA model can only extract the linear information in the data, but the valuable nonlinear information in the data is not processed, while the LSTM model can extract the nonlinear components in the data. This suggests that, ARIMA model is suitable for relatively stable series, while LSTM model is suitable for relatively unstable series [5].

However, in practical application, a single prediction model or method has different emphasis on extracting time series data information, so its prediction accuracy is still insufficient when dealing with complex and dynamic time series. Compared with a single prediction model, combined prediction model can effectively reduce system risks while ensuring better prediction performance, thus becoming the mainstream trend in time series prediction research [6].

In recent years, the combinatorial model constructed based on the idea of decomposition and integration decomposes the original sequence to reduce the sequence complexity and obtain sequences with simpler structure, more stable changes and stronger regularity. The accuracy of time series prediction is improved by modeling the decomposed sequence. Empirical Mode Decomposition (EMD) is an adaptive decomposition method for nonlinear and non-stationary signals proposed by Huang and his co-authors in 1998 [7]. It decomposes the original time series into multiple Intrinsic mode functions (IMF) in different time scales and a residual signal (RES). Not only can it be directly applied to nonlinear and non-stationary time series, but also can reveal the changes of different time scales contained in time series [8]. In 2022, An [9] used the Back-Propagation Neural Network (BPNN) model based on EMD to predict incidence of Acquired Immune Deficiency Syndrome (AIDS). First, EMD method was used to decompose the original sequence into four relatively stable IMFs and a residual signal, and then all the decomposition results were respectively established BPNN models and summed to obtain the EMD-BPNN predicted value. Compared with the prediction results of single BPNN and ARIMA, it was found that the prediction effect of EMD-BPNN hybrid model is superior to the above models, that was, the hybrid model improved the prediction accuracy. In 2021, Wang [10] proposed a short-term generation combination forecasting model based on EMD-LSTM-ARMA. Firstly, the normalized IMF1 and IMF2 are input into the designed LSTM network to model and predict, then the IMF3 is modeled and predicted by the ARMA model, and then a low-frequency component is reconstructed from IMF4, IMF5 and residual components. The empirical results show that EMD-LSTM-ARMA combined forecasting model can produce higher forecasting accuracy than single model.

In order to further improve the prediction performance, this paper used EMD to break down the original sequence into several subsequences, and chose the sequences meeting the stationarity requirements to build an ARMA model and the unstable sequences to build an LSTM model after judging the stationarity of the decomposed sequences. On this basis, the EMD-ARMA-LSTM hybrid prediction model was constructed to provide a theoretical foundation for forecasting the epidemic trend of tuberculosis and developing prevention and control programs.

## Material and methods

### Data sources

The reported incidence data of pulmonary tuberculosis used in this study were from the National Public Health Science Data Center (https://www.phsciencedata.cn/), and a total of 132 months of reported incidence data of tuberculosis (per 100 000) from 2008 to 2018 were downloaded, the reported incidence rates of tuberculosis in China from January 2008 to December 2017 were used as the training set to predict the reported incidence of pulmonary tuberculosis in the next 3 months, 6 months, 9 months and 12 months.

### Empirical modal decomposition (EMD)

EMD is a new signal decomposition method. Compared with traditional signal decomposition methods, it completely gets rid of the restriction of basis function and can decompose any signal (time series) in theory [11]. The core idea of this algorithm is to decompose complex original data into a finite number of IMFs with different scales, stationarity and periodic volatility characteristics and a residual signal representing the overall trend of the original signal. Therefore, it has good adaptability to nonlinear and non-stationary sequences. The IMF should meet the following two conditions: (1) The number of extremes does not differ from the number of zeros by more than 1. (2) At any point in an envelope represented by a local maximum and an envelope represented by a local minimum, the average of both is zero. The decomposition steps are as follows [12]:

(1) All maximum points and minimum points on the original tuberculosis sequence are calculated;

(2) By cubic spline interpolation method, the local maximum and local minimum points are constructed into the upper and lower envelope(${e}_{t\left(\mathrm{min}\right)}$、${e}_{t\left(\mathrm{max}\right)}$), and then the average value of the two envelope lines is calculated:

1 $${m}_{t}\text{=}\left({e}_{t\left(\mathrm{min}\right)}+{e}_{t\left(\mathrm{max}\right)}\right)/2$$

Subtract ${m}_{t}$ from the original signal:

2 $${d}_{t}\text{=}{X}_{t}-{m}_{t}$$

Determine whether ${d}_{t}$ meets the conditions of IMF, if so, ${d}_{t}$ at this time is the first IMF component obtained by decomposition, denoted as $IM{F}_{t}^{1}={d}_{t}$; If it is not satisfied, we need to take ${d}_{t}$ as the new original signal ${X}_{t}$ and repeat steps (1) and (2) until it is satisfied.

(3) The original sequence and the newly obtained intrinsic mode function component are calculated to obtain the residual components after the first decomposition.

3 $${r}_{t}={X}_{t}-IM{F}_{t}^{1}$$

Repeat step (1) until the loop stops. The original sequence at this point can be expressed as:

4 $${X}_{t}=\sum_{i=1}^{N}{IMF}_{t}^{i}+{r}_{n}(t)$$

where ${IMF}_{t}^{i}$ is the ith intrinsic mode function component, and ${r}_{n}(t)$ represents the nth residual sequence.

### Seasonal Autoregressive Integrated Moving Average (SARIMA)

ARIMA is a time series forecasting method proposed by Box, an American statistician, and Jenkins, a British statistician. The basic idea is to regard the data formed by the predicted object as a random sequence, use the corresponding mathematical model to describe the autocorrelation in the sequence and predict future values from potential relationships between past and present values of a sequence [13]. It has two forms: non-seasonal ARIMA model and seasonal ARIMA model, and its expressions are ARIMA (p, d, q) and ARIMA (p, d, q) (P, D, Q) s. Where, p and P represent the autoregressive order and seasonal autoregressive order respectively. d and D are difference order and seasonal difference order respectively. q and Q are the moving average order and seasonal moving average order, and s is the cycle length [14]. The specific modeling process of ARIMA model is as follows: (1) Stationarity test: Autocorrelation Function (ACF) plot, and Augmented Dickey-fuller (ADF) test were used to comprehensively judge whether the time series data was stable. If it was non-stable, d or D-order difference processing was required. (2) Ljung-Box test: Ljung-Box test was performed on the sequence, if the p value was less than the signifcance level, the sequence had no randomness. Modeling can be continued if the sequence was non-random. (3) Model identification and order determination: Python Grid Search was used to automatically fit the SARIMA model. (4) Model selection: according to the minimum Akaike information criterion (AIC), the optimal model was selected. (5) Model test: the success of model fitting was judged by the residual white noise test. If the residual sequence was random, the model is fitted successfully. (6) Prediction: use the constructed model to make predictions.

### Long-short term memory (LSTM) model

**Fig. 1:**
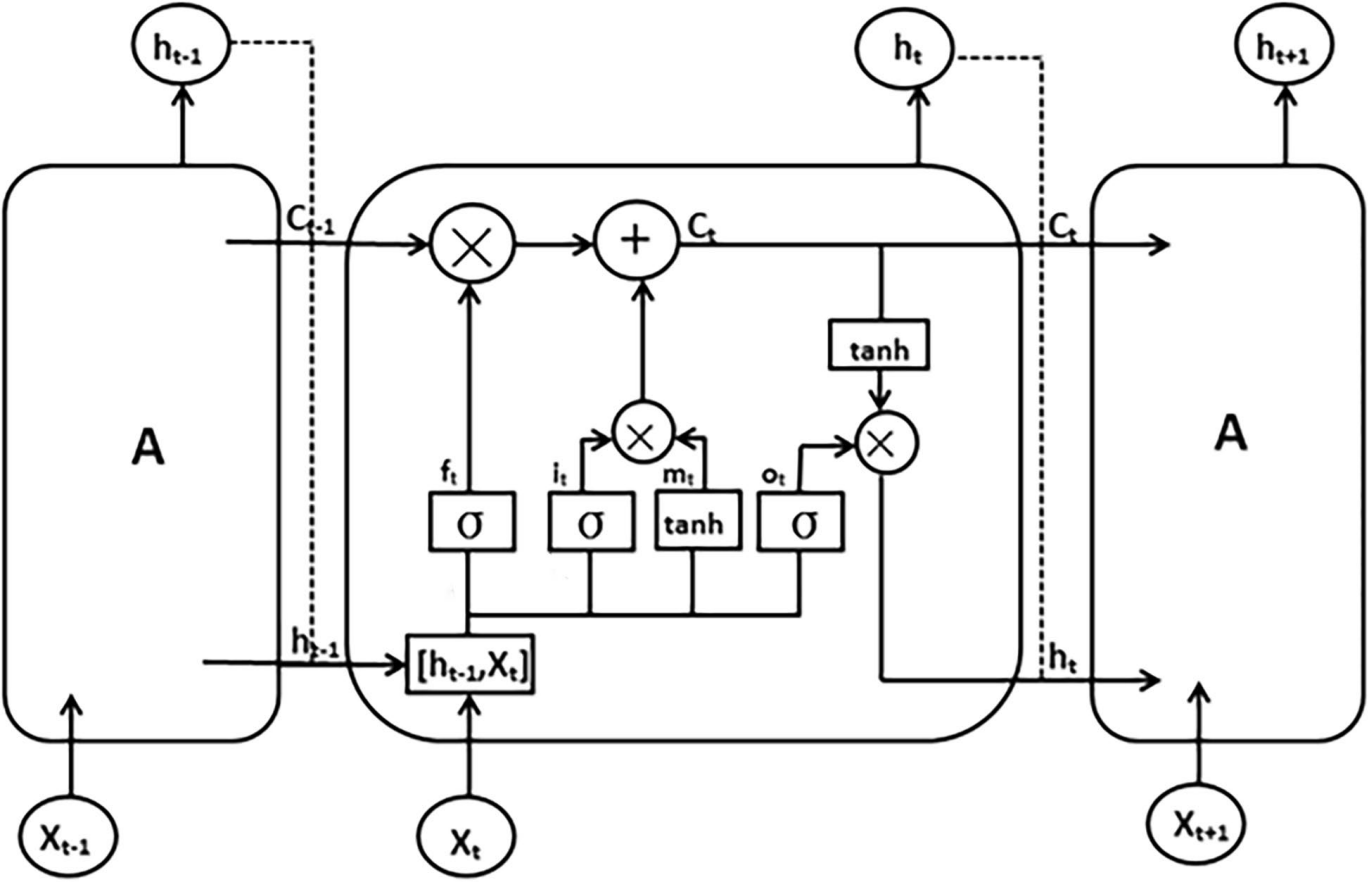
LSTM network unit structure

LSTM network is a kind of Recurrent Neural Networks (RNN) with special network structure proposed by Sepp Hochreiter and Jurgen Schmidhuber in 1997 for the gradient dispersion problem of RNN model [15]. It is characterized by introducing memory unit in each neuron of the hidden layer and solving the contradiction problem of input and output weights through input and output gates, so that it can make more effective use of long-distance time series data. Thus, the long-term dependence problem in traditional RNN model training was overcome [16]. LSTM unit structure is also known as memory unit (A). Its structure was shown in Fig. 1, including three gated structures [17], namely "forget Gates(${f}_{t}$)", " input Gates(${i}_{t}$)" and "output Gates(${o}_{t}$)", these three gating structures can selectively control passage of information [18], and also include the cell state ${C}_{t}$ representing long-term memory, and the candidate state ${m}_{t}$ waiting to be deposited in long-term memory [19]. The calculation formula of each calculation gate is as follows [20]:

5 $${f}_{t}=\sigma \left({W}_{fh}{h}_{t-1}+{W}_{fx}{x}_{t}+{b}_{f}\right)$$

6 $${i}_{t}=\sigma \left({W}_{ih}{h}_{t-1}+{W}_{ix}{x}_{t}+{b}_{i}\right)$$

7 $${o}_{t}=\sigma \left({W}_{oh}{h}_{t-1}+{W}_{ox}{x}_{t}+{b}_{o}\right)$$

8 $${m}_{t}=\mathrm{tanh}\left({W}_{mh}{h}_{t-1}+{W}_{mx}{x}_{t}+{b}_{m}\right)$$

9 $${C}_{t}={f}_{t}{C}_{t-1}+{i}_{t}{\mathrm{m}}_{t}$$

10 $${h}_{t}={o}_{t}\mathrm{tanh}\left({C}_{t}\right)$$

In the above equation, $W$ is the weight matrix connecting the two layers, $\upsigma$ is the sigmoid activation function, $b$ is the corresponding offset item and the tanh function represents the feed-forward network layer of the hyperbolic tangent function. ${h}_{t-1}$ represents the output at time $t-1$, and ${X}_{t}$ represents the input at time $t$.

The construction process of LSTM model is as follows [14]:

1. Data preprocessing, including normalization and reconstruction of data;
2. The original sequence is transformed into three-dimensional data;
3. The original sequence was divided into training set and test set;
4. Adjust model parameters.

### EMD-SARIMA combined model

The pulmonary tuberculosis sequence was decomposed by EMD method to obtain a group of IMFs and a residual signal. The decomposed IMF components contain partial characteristic signals of different time scales of the original signals, and the EMD method completely throw away the constraint of the basis function, and has good compatibility for various signals. EMD-SARIMA model is a combination model based on the idea of "decomposition before integration" [21]. The specific steps are as follows:

1. The original pulmonary tuberculosis signal was decomposed into multiple IMFs and a residual signal using the EMD method;
2. Each IMF component and residual signal were predicted by the corresponding SARIMA model;
3. According to the completeness of EMD and the orthogonality of IMF, the predicted values of the above parts are summed and reconstructed [22] to get the final results.

### EMD-LSTM combined model

Due to the characteristics of its internal structure, LSTM model can realize long-term learning of dependent information [11]. The specific steps of EMD-LSTM are as follows:

1. The original pulmonary tuberculosis signal was decomposed by EMD method to obtain finite IMFs and a residual signal representing the overall trend of the original signal;
2. Each IMF component and residual signal were predicted by the corresponding LSTM;
3. The predicted value of the original signal was obtained by superposition of the predicted value of each decomposition sequence.

### EMD-ARMA-LSTM Combined Model

**Fig. 2:**
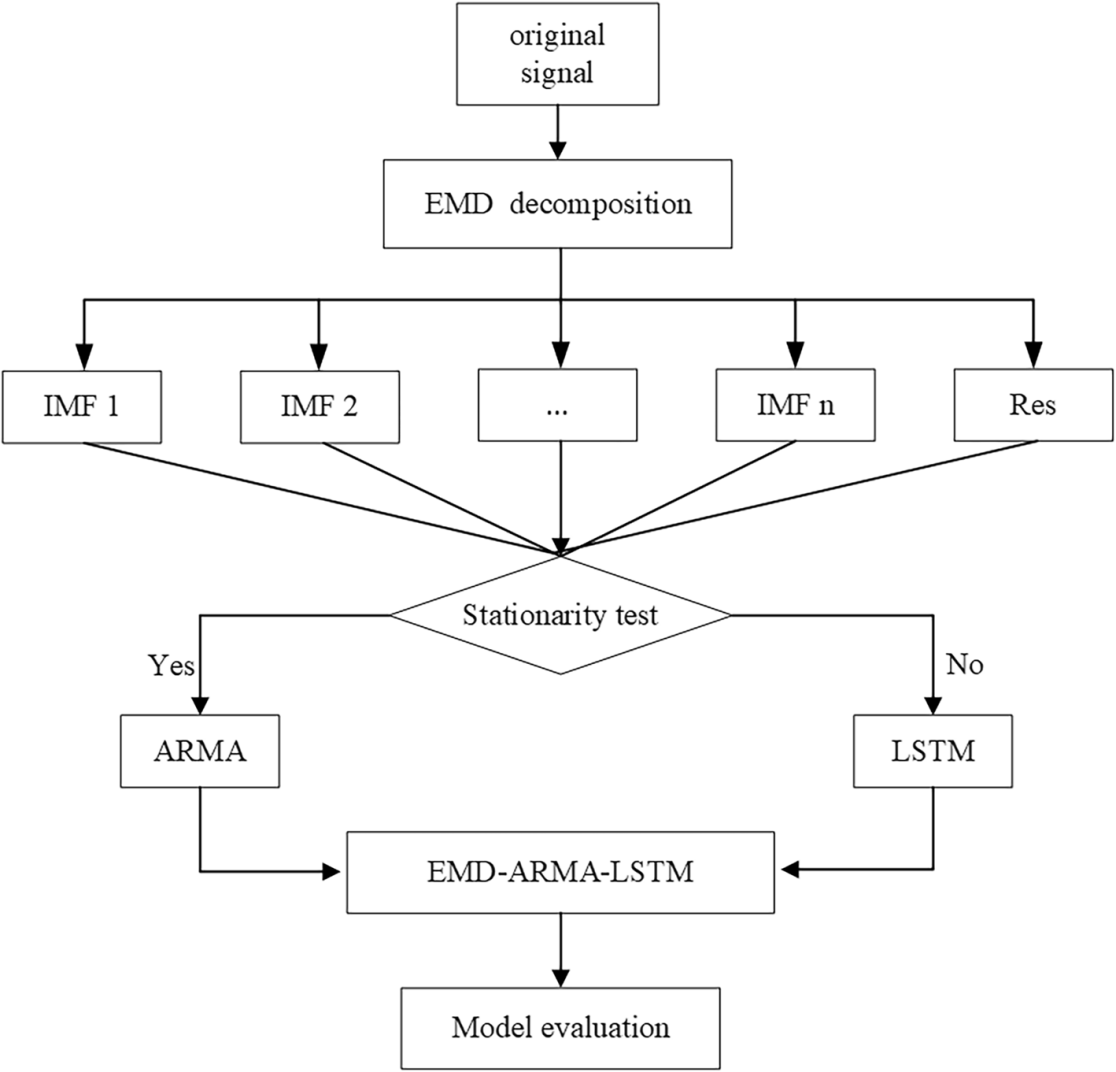
EMD-ARMA-LSTM modeling process

The results of single prediction model based on decomposition show that the subsequences obtained by EMD method all have stationary and non-stationary sequences. In view of the shortage of direct modeling without feature analysis after the decomposition of the above model, the stationarity of the decomposed sequences was judged, and, and then chose the sequences meeting the stationarity requirements to build an ARMA model and the unstable sequences to build an LSTM model. On this basis, the EMD-ARMA-LSTM hybrid prediction model was constructed. The modeling process was shown in Fig. 2:

### Model effect evaluation

The model performance evaluation of continuous data mainly depends on the difference between the predicted value and the real value. The smaller the value is, the better the model prediction effect will be. Mean Squared Error (MSE), Mean Absolute Error (MAE), Root Mean Squared Error (RMSE) and Mean Absolute Percentage Error (MAPE) were used to compare the predictive performance of each model.

11 $$MAE=\frac{1}{N}\sum_{k=1}^{N}\left|{X}_{k}-{\widehat{X}}_{k}\right|$$

12 $$MSE=\frac{1}{N}\sum_{k=1}^{N}{\left({X}_{k}-{\widehat{X}}_{k}\right)}^{2}$$

13 $$RMSE=\sqrt{\frac{1}{N}\sum_{k=1}^{N}{\left({X}_{k}-{\widehat{X}}_{k}\right)}^{2}}$$

14 $$MAPE=\frac{100\mathrm{\%}}{n}\sum_{i=1}^{n}\left|\frac{{\widehat{X}}_{k}-{X}_{k}}{{X}_{k}}\right|$$

where, ${X}_{k}$ represents the real value at the moment $k$, ${\widehat{X}}_{k}$ represents the predicted value of each model, and $N$ represents the sample size during the test.

### Statistical analysis

Excel software version 2021 was used for data collection and collation, Anaconda software version 4.10.3 was used to establish the SARIMA model and the LSTM model. MATLAB software version 2022 was used for EMD.

## Results

### Time distribution of pulmonary tuberculosis in China

**Fig. 3:**
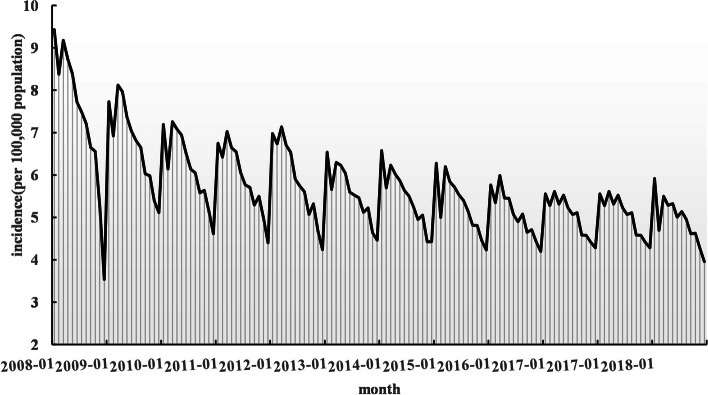
Time series of tuberculosis incidence from 2008 to 2018

The time series of the reported incidence of pulmonary tuberculosis in China from January 2008 to December 2018 was shown in Fig. 3. It can be seen that the reported incidence of pulmonary tuberculosis in China presents a decreasing trend year by year and has obvious seasonality, with two apparent epidemic peaks in January and March of each year, which were close to the results of previous research [23].

#### EMD

**Fig. 4:**
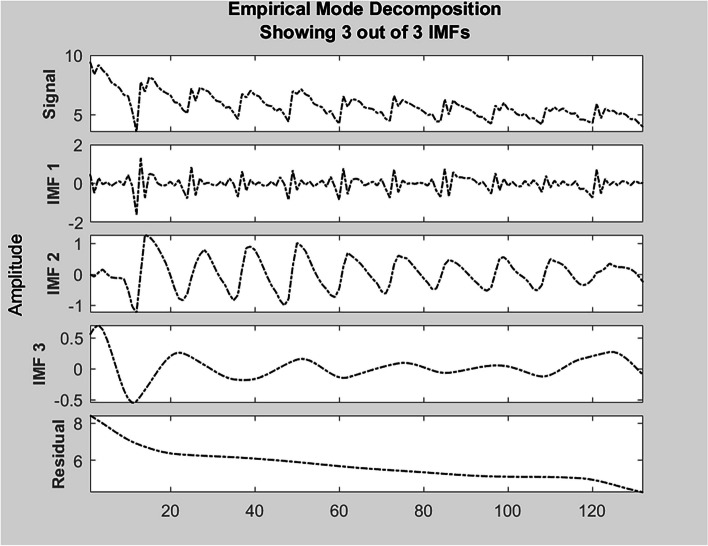
Primary signal of pulmonary tuberculosis and decomposition of EMD

The original sequence of tuberculosis was decomposed by EMD, and three IMFs and a residual signal were obtained, among which IMF1 had the highest frequency and represented the high frequency component of tuberculosis signal, residual signal is the lowest frequency signal and represents the trend in the pulmonary tuberculosis signal. The original signal of pulmonary tuberculosis and each decomposition sequence after EMD decomposition were shown in Fig. 4.

### SARIMA Model

**Fig. 5:**
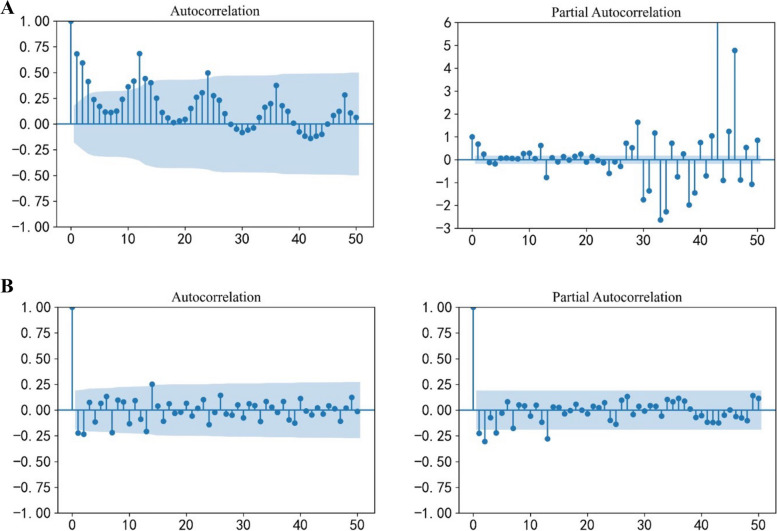
Autocorrelation and partial autocorrelation plots of original sequence: (**A**) is before difference and (**B**) is after difference

The SARIMA model was constructed for the original sequence, three IMFs and a residual signal respectively. The original sequence, IMF1, IMF3 and residual signal were non-stationary sequences, so difference processing was carried out to make them stationary, all the sequences became stationary after d or D-order difference. IMF2 was a stationary sequence. Taking the original sequence as an example, the ACF/PACF figure before and after difference is shown in Fig. 5. All the adjusted sequences and IMF2 were non-white noise (Table 1). The d and D of the original sequence, IMF3 and residual signal were determined by the number of differences in the sequence. According to previous literature experience, the values of p, q, P and Q ranged from 0 to 2, and the SARIMA model was automatically fitted by Python grid search [24]. According to AIC minimum principle, the optimal models were determined as follows: Original sequence: SARIMA (2,1,0) (1,1,2) _12_; IMF1: SARIMA (2,0,2) (0,1,2) _12_; IMF2: ARMA (2,2); IMF3: SARIMA (2,1,1) (0,0,1) _12_; Residual signal: ARIMA (2,2,1). Ljung-Box test was performed on the residual sequence of the models, the results showed that the p value was more than the significance level and the sequences were random (Table 2). The above models were used to predict the corresponding IMF and residual signal, and the predicted values were integrated in the form of direct summation to obtain the final forecast results of the EMD-SARIMA model.

**Table 1:** Unit root test and white noise test results after difference of each sequence

Sequence	ADF		Box-Ljung	
	*t*	*P*	*χ*^*2*^	*P*
Original sequence	-5.690	< 0.001	12.250	0.006
IMF1	-3.581	0.006	9.778	0.021
IMF2	-4.003	0.001	95.781	< 0.001
IMF3	-4.013	0.001	190.242	< 0.001
Res	-4.077	< 0.001	195.374	< 0.001

**Table 2:** Residual white noise test results of each prediction model

Model	Box-Ljung	
	*χ*^*2*^	*P*
Original sequence—SARIMA(2,1,0)(1,1,2)_12_	0.030	0.860
IMF1—SARIMA(2,0,2)(0,1,2)_12_	0.240	0.630
IMF2—ARMA(2,2)	0.010	0.930
IMF3—SARIMA(2,1,1)(0,0,1)_12_	0.050	0.820
Res—ARIMA(2,2,1)	1.740	0.190

### LSTM Model

Since appropriate model parameters have a great impact on the prediction performance, the last 12 data of the training set were used as verification sets to adjust the model parameters. Due to the obvious periodicity of the original sequence of pulmonary tuberculosis, the window length of the LSTM network was set to 12, that was, the number of nodes in the input layer was set to 12. The following one-month data was used as the output for prediction, and the number of nodes in the output layer was set to 1. Since the number of hidden layer nodes has a great impact on the model accuracy, the empirical formula $M=\sqrt{m+n}+a (a=1\sim 10)$ [25] was used to determine the range of node number M. In this paper, the number of hidden layer nodes was first determined under the condition that the number of hidden layers was fixed as 1. The M calculated by the experimental formula was 5–13. When the number of hidden layer nodes was set to 13, the LSTM had the smallest error value (Table 3).

**Table 3:** Influence of the number of hidden layer nodes on the fitting performance of the model

Nodes number	5	6	7	8	9	10	11	12	**13**
MSE	0.022	0.023	0.029	0.017	0.020	0.231	0.020	0.029	**0.017**
MAE	0.116	0.129	0.142	0.117	0.116	0.125	0.118	0.141	**0.104**
RMSE	0.150	0.151	0.169	0.132	0.140	0.152	0.143	0.171	**0.130**
MAPE	0.022	0.025	0.028	0.023	0.022	0.024	0.023	0.027	**0.020**

When the number of hidden layer nodes was fixed at 13, the experiment was carried out with the number of hidden layer layers 1–4, when the number of hidden layers was set to 1, the error value of LSTM was the lowest (Table 4).

**Table 4:** Influence of the number of hidden layers on the fitting performance of the model

Layers number	**1**	2	3	4
MSE	**0.017**	0.023	0.027	0.027
MAE	**0.104**	0.122	0.136	0.127
RMSE	**0.130**	0.152	0.163	0.163
MAPE	**0.020**	0.023	0.026	0.024

To sum up, this paper finally set the window length as 12, the number of hidden layers as 1, and the number of hidden layer nodes as 13, and fixed the number of seeds of the model, selected 1000 iterations, set batch size as 32, and used Adam optimizer to predict the model.

### Comparative analysis of models

**Fig. 6:**
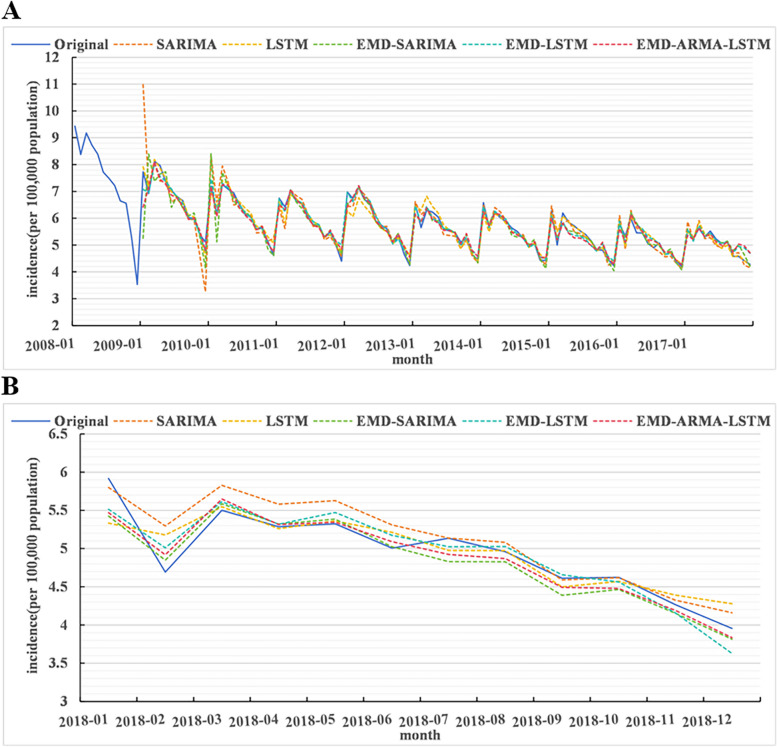
Comparative chart for predicting the incidence of tuberculosis in the next year. **A** was the fitting period, and (**B**) was the test period

In this paper, the pulmonary tuberculosis sequence was transformed into a series of relatively stable subsequences by EMD decomposition. Then, the decomposition-based single model and the decomposition-based hybrid model were established respectively to predict the incidence trend in the next year, and compared with the prediction results of the undecomposed single model, as shown in Fig. 6. Additionally, we built corresponding prediction models using the next 3 months, 6 months, and 9 months as the test period in order to assess the robustness of the results.

1. Comparing decomposition-based single model with undecomposed single model, it was found that: when predicting the incidence trend in the next year, compared with SARIMA model, the MSE, MAE, RMSE and MAPE of EMD-SARIMA decreased by 39.3%, 19.0%, 22.1% and 19.8%, respectively. The MSE, MAE, RMSE and MAPE of EMD-LSTM were reduced by 40.5%, 12.8%, 22.9% and 12.7%, respectively, compared with the LSTM model. Furthermore, the performance of the model were consistent when predicting the incidence trend in the next 3 months, 6 months and 9 months (Table 5).
2. Comparing the decomposition-based hybrid model with the decomposition-based single model, it was found that: when predicting the incidence trend in the next year, compared with EMD-SARIMA model, the MSE, MAE, RMSE and MAPE of EMD-ARMA-LSTM model decreased by 21.7%, 10.6%, 11.5% and 11.2%, respectively. The MSE, MAE, RMSE and MAPE of EMD-ARMA-LSTM were reduced by 16.7%, 9.6%, 8.7% and 12.3%, respectively, compared with EMD-LSTM model. Furthermore, the performance of the model were consistent when predicting the incidence trend in the next 3 months, 6 months and 9 months (Table 6).

**Table 5:** Comparison of the decomposition-based single model and undecomposed single model

Time	Model	MSE		MAE		RMSE		MAPE	
		value	rate(%)	value	rate(%)	value	rate(%)	value	rate(%)
3 months	SARIMA	0.1615	-	0.3489	-	0.4018	-	0.0692	-
	EMD-SARIMA	**0.0912**	**43.5**	**0.2439**	**30.1**	**0.3019**	**24.9**	**0.0439**	**36.6**
	LSTM	0.1553	-	0.3273	-	0.3940	-	0.0614	-
	EMD-LSTM	**0.0804**	**48.2**	**0.2579**	**21.2**	**0.2835**	**28.0**	**0.0501**	**18.4**
6 months	SARIMA	0.1261	-	0.3250	-	0.3551	-	0.0636	-
	EMD-SARIMA	**0.0465**	**63.1**	**0.1413**	**56.5**	**0.2156**	**39.3**	**0.0256**	**59.7**
	LSTM	0.1039	-	0.2312	-	0.3223	-	0.0440	-
	EMD-LSTM	**0.0390**	**62.5**	**0.1509**	**34.7**	**0.1975**	**38.7**	**0.0288**	**34.5**
9 months	SARIMA	0.0858	-	0.2334	-	0.2929	-	0.0458	-
	EMD-SARIMA	**0.0488**	**43.1**	**0.1674**	**28.3**	**0.2208**	**24.6**	**0.0320**	**30.1**
	LSTM	0.0763	-	0.1820	-	0.2763	-	0.0347	-
	EMD-LSTM	**0.0422**	**44.7**	**0.1735**	**4.7**	**0.2055**	**25.6**	**0.0336**	**3.2**
12 months	SARIMA	0.0682	-	0.1976	-	0.2612	-	0.0399	-
	EMD-SARIMA	**0.0414**	**39.3**	**0.1600**	**19.0**	**0.2035**	**22.1**	**0.0320**	**19.8**
	LSTM	0.0654	-	0.1813	-	0.2558	-	0.0371	-
	EMD-LSTM	**0.0389**	**40.5**	**0.1581**	**12.8**	**0.1972**	**22.9**	**0.0324**	**12.7**

**Table 6:** Comparison of the decomposition-based hybrid model and the decomposition-based single model

Time	Model	MSE		MAE		RMSE		MAPE	
		value	rate(%)	value	rate(%)	value	rate(%)	value	rate(%)
3 months	EMD-SARIMA	0.0912	-	0.2439	-	0.3019	-	0.0439	-
	EMD-ARMA-LSTM	**0.0700**	**23.2**	**0.2406**	**1.4**	**0.2645**	**12.4**	**0.0447**	**-1.8**
	EMD-LSTM	0.0804	-	0.2579	-	0.2835	-	0.0501	-
	EMD-ARMA-LSTM	**0.0700**	**12.9**	**0.2406**	**6.7**	**0.2645**	**6.7**	**0.0447**	**10.8**
6 months	EMD-SARIMA	0.0465	-	0.1413	-	0.2156	-	0.0256	-
	EMD-ARMA-LSTM	**0.0355**	**23.7**	**0.1283**	**9.2**	**0.1885**	**12.6**	**0.0239**	**6.6**
	EMD-LSTM	0.0390	-	0.1509	-	0.1975	-	0.0288	-
	EMD-ARMA-LSTM	**0.0355**	**9.0**	**0.1283**	**15.0**	**0.1885**	**4.6**	**0.0239**	**17.0**
9 months	EMD-SARIMA	0.0488	-	0.1674	-	0.2208	-	0.0320	-
	EMD-ARMA-LSTM	**0.0321**	**34.2**	**0.1438**	**14.1**	**0.1792**	**18.8**	**0.0275**	**14.1**
	EMD-LSTM	0.0422	-	0.1735	-	0.2055	-	0.0336	-
	EMD-ARMA-LSTM	**0.0321**	**23.9**	**0.1438**	**17.1**	**0.1792**	**12.8**	**0.0275**	**18.2**
12 months	EMD-SARIMA	0.0414	-	0.1600	-	0.2035	-	0.0320	-
	EMD-ARMA-LSTM	**0.0324**	**21.7**	**0.1430**	**10.6**	**0.1800**	**11.5**	**0.0284**	**11.2**
	EMD-LSTM	0.0389	-	0.1581	-	0.1972	-	0.0324	-
	EMD-ARMA-LSTM	**0.0324**	**16.7**	**0.1430**	**9.6**	**0.1800**	**8.7**	**0.0284**	**12.3**

## Discussion

In the past two decades, great progress had been made in the prevention and control of pulmonary tuberculosis, but pulmonary tuberculosis is still a major public health problem endangering people's health [26], and "precise prevention" is the key to the current prevention and control of tuberculosis [27]. Therefore, the timely understanding of tuberculosis epidemic trend and the establishment of accurate tuberculosis prediction model can provide scientific basis for the formulation of disease prevention and control policies.

In recent years, most predictions of infectious diseases are based on the original time series model. Studies have shown that, compared with a single prediction model, the combination model built based on the decomposition and integration idea can reduce the complexity of the sequence and effectively improve the prediction performance of the model by decomposing the original sequence. Comparing the decomposition-based single model with the undecomposed single model, it was found that the prediction performance of the decomposition-based single model was better than that of the undecomposed single model, which was mainly due to the decomposition of the initial sequence, so as to obtain relatively simple, stable and regular subsequence, which reduced the difficulty of model and improved the accuracy of prediction. Secondly, in view of the limitation of using only a single model for prediction in the analysis process, this paper attempted to use SARIMA model for stationary series and LSTM model for non-stationary series to establish a decomposition-based hybrid model. Compared with the decomposition-based corresponding single model, it was found that constructing the combined model can improve prediction performance of the model, indicating that selecting the appropriate model according to the subsequence characteristics was beneficial to improve the performance of the model. In conclusion, compared with other models, the combined model of EMD-ARMA-LSTM adopted in this study can improve the prediction accuracy more effectively, make a more accurate and reasonable prediction of pulmonary tuberculosis, and provide a theoretical basis for the prediction of tuberculosis epidemic trend and the formulation of prevention and control policies.

The innovation of this paper is to build an EMD-ARMA-LSTM hybrid model based on the idea of decomposition before integration, and use the existing incidence data of tuberculosis to predict the incidence trend of this infectious disease in the next year, and achieve good results. In addition, we ensured the robustness of the model by changes in results over the next 3 months, 6 months, and 9 months as test period. The model is not only suitable for predicting an infectious disease such as tuberculosis, but can also be extended to other datasets such as hand, foot and mouth disease and influenza. Therefore, this study has certain applicability in the field of epidemiology, which can not only improve people's attention to infectious diseases through the prediction of future incidence, but also help relevant departments to formulate relevant prevention and control policies.

## Conclusion

1. The reported incidence of tuberculosis in China from 2008 to 2018 showed a decreasing trend year by year, with obvious seasonality, with two obvious epidemic peaks in January and March each year.
2. The prediction performance of EMD-SARIMA model was better than that of SARIMA model, and that of EMD-LSTM model was better than that of LSTM model. This suggested that the prediction performance of single model based on EMD decomposition was better than that of undecomposed single model.
3. The predictive performance of EMD-ARMA-LSTM model was better than that of EMD-SARIMA model and EMD-LSTM model. This suggested that the prediction performance of the combined model using the advantages of different models was better than that of decomposition-based single model.

## Data Availability

The datasets generated and analysed during the current study are available in the [the National Public Health Science Data Center] repository, [https://www.phsciencedata.cn/].
